# Reactivity Tracking of an Enzyme Progress Coordinate

**DOI:** 10.1021/acs.jpclett.3c01464

**Published:** 2023-08-04

**Authors:** Wei Li, Meghan Kohne, Kurt Warncke

**Affiliations:** † Department of Physics, Emory University, Atlanta, Georgia 30322, United States

## Abstract

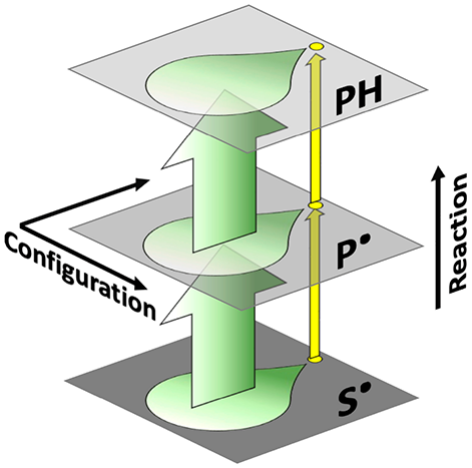

The reactivity of individual solvent-coupled protein configurations is used to track and resolve the progress coordinate for the core reaction sequence of substrate radical rearrangement and hydrogen atom transfer in the ethanolamine ammonia-lyase (EAL) enzyme from *Salmonella enterica*. The first-order decay of the substrate radical intermediate is the monitored reaction. Heterogeneous confinement from sucrose hydrates in the mesophase solvent surrounding the cryotrapped protein introduces distributed kinetics in the non-native decay of the substrate radical pair capture substate, which arise from an ensemble of configurational microstates. Reaction rates increase by >10^3^-fold across the distribution to approach that for the native enabled substate for radical rearrangement, which reacts with monotonic kinetics. The native progress coordinate thus involves a collapse of the configuration space to generate optimized reactivity. Reactivity tracking reveals fundamental features of solvent–protein-reaction configurational coupling and leads to a model that refines the ensemble paradigm of enzyme catalysis for strongly adiabatic chemical steps.

The canonical resting, intermediate, and transition states of enzymes comprise a distribution of protein conformations.^1−3^ The progress coordinate for the catalytic cycle has been described as a stochastic, thermal equilibration of reactant(s) and protein conformations within a coupled network of conformational states,^4,5^ that establish the geometry, electrostatic environment, and hydrogen bonding patterns for each step of the reaction sequence.^3,6^ This ensemble view emerges primarily from high-resolution protein structure-monitoring techniques,^2,3,7,8^ augmented by atomic-level mechanistic descriptions.^5,9^ These methods are sensitive to conformational changes that are involved in reactant or cofactor binding and release, or excursions of protein segments, such as loops,^2,3,7,8^ on the micro- to millisecond time scale and the few to several Ångstroms spatial scale at ambient temperature (*T*). The conformational heterogeneity is proposed to persist through the different intermediate states of the catalytic cycle, including the apoenzyme,^2,7,10^ and correlations with function extend globally from active site to protein surface, and into the solvent.^7,11^ As a contrast and complement to *structure*, we use chemical *reactivity* as the parameter for discerning and quantifying the evolution of function along the progress coordinate for the enzyme process. “Reactivity tracking” measures the first-order rate constants of a particular reaction for the different configurations or states that comprise the progress coordinate. This includes the native state, which is specifically enabled for the particular reaction, as well as nearby to distant neighbor configurational states. The progress coordinate is thus parametrized as a function of reactivity, which is a higher-order representation of structure. The combination of measured state population and corresponding rate constant information provides a unique perspective that reveals fundamental features of the catalysis of adiabatic chemical reactions by enzymes. Here, the substrate radical reactivities of individual cryotrapped microstates that comprise the progress coordinate through the core sequence of the adenosylcobalamin (coenzyme B_12_)-dependent ethanolamine ammonia-lyase (EAL; EC 4.3.1.7)^12,13^ from *Salmonella enterica* serovar Typhimurium are resolved.

Time-resolved reactions of cryotrapped, out-of-equilibrium states, triggered by temperature-step annealing or photoexcitation, have previously provided insight into the molecular mechanisms of protein processes. Building on pioneering cryoreduction and thermal annealing studies^14^ of heme proteins, Hoffman and co-workers determined metallocenter structures, reaction kinetics, and ^1^H/^2^H hydrogen isotope effects at 253 K, by using electron paramagnetic resonance (EPR) spectroscopy, to define the central, three-state sequence involved in stoichiometric accumulation of reducing equivalents and dihydrogen evolution preceding dinitrogen binding and reduction, in nitrogenase.^15^ Kinetics of the long-range electron transfer reaction between the photogenerated reduced quinone and oxidized bacteriochlorophyll dimer in cryotrapped and annealed bacterial photosynthetic reaction center (RC) protein were characterized at <100 K.^16^ Small molecule (CO, O_2_) migration through the protein interior and heme rebinding following low-temperature photolysis in myoglobin (Mb) provide a global perspective on contributions of solvent-coupled protein configurations and fluctuations to function.^17,18^ In these disparate systems, rate constants measured at low temperatures typically display a distribution of values that can be characterized by a power-law, representing a summation of first-order, exponential processes.^19^ The cause is low-temperature stabilization of the Boltzmann distribution of reactant and protein microscopic configurations on the measurement time-scale. The low-temperature measurements thus reveal the native microscopic configurations and changes of the system that are averaged at physiological temperatures by rapid interconversion (exchange) among the microscopic states to give the detected exponential kinetics and mean rate constants.^20^ We extend this type of measurement to address adiabatic, covalent bond-making and bond-breaking reactions in enzymes.

The core adiabatic reaction sequence in EAL (Figure 1A) involves substrate radical rearrangement (RR; rate-limiting C–N bond cleavage followed by 1,2-migration and reattachment of the amino group),^12,21^ to form the product radical, followed by hydrogen atom (H^•^) transfer (HT; donor, C5′-methyl group of 5′-deoxyadenosine; acceptor, product radical) to form products. Radical pair recombination (RPR) subsequently reforms the resting diamagnetic cobalamin cofactor.^22−26^ During steady-state turnover, the cob(II)alamin-substrate radical pair state (**S**^•^; defined as a “canonical state” in the enzyme cycle by the distinct active site reactant species, 2-aminoethan-1-ol-1-yl radical) accumulates in EAL, because RR is the slowest step in the cycle, and is cryotrapped in high yield.^27^ EAL frozen in aqueous solutions in the presence of added substrate aminoethanol (100 mM, 0.6% v/v) harbors an intact protein hydration layer surrounded by an aqueous-aminoethanol mesodomain (Figure 1B),^28,29^ that maintains the native protein structure^30,31^ and reaction pathway of the substrate radical for *T* ≥ 220 K. Reactions of the cryotrapped substrate radical in frozen solution are initiated by a *T*-step and monitored by using time-resolved, full-spectrum EPR spectroscopy (Figure 1C).^24,26,27^ At *T* = 220 K, a free energy barrier rises and partitions the canonical **S**^•^ state, and for *T* < 220 K monoexponential decay reactions proceed from two “substates,” **S**_**1**_^•^ and **S**_**2**_^•^ (substates of the canonical state are defined as having distinct decay kinetics from the same reactant, which is here the 2-aminoethan-1-ol-1-yl radical) (Figure 1A). The **S**_**1**_^•^ (slow decay; observed rate constant, *k*_obs,s_) and **S**_**2**_^•^ (fast decay; *k*_obs,f_; *k*_obs,f_/*k*_obs,s_ ≈ 10) substates are sequential intermediate states,^24^ distinguished by protein configurations that have two distinct functions:^25,26^ (1) **S**_**1**_^•^ captures the nascent substrate radical directly after its creation by radical pair separation, and (2) **S**_**2**_^•^ enables the substrate radical for reaction to the product radical. At *T* < 220 K, active sites cryotrapped in **S**_**2**_^•^ continue to react along the native path, while sites cryotrapped in **S**_**1**_^•^ follow a non-native path. For both pathways, reaction is rate-limited by RR (for natural isotopic abundance aminoethanol; denoted as ^1^H-substrate) or a combination of RR and HT (1,1,2,2-^2^H-labeled aminoethanol; denoted as ^2^H-substrate).^12,26,32^ HT is partially rate-determining for the ^2^H-substrate, because turnover on the ^2^H-substrate during cryotrapping leads to incorporation of ^2^H into the C5′-carbon of 5′-deoxyadenosine (Ad-CH_3_, Figure 1A).^32^ The ^2^H isotope [mass ratio, *m*(^2^H)/*m*(^1^H) = 2] slows the hydrogen atom transfer from Ad-CH_3_ to the C2 center of the product radical, relative to the transfer of ^1^H.^12,26,32^

**Figure 1:**
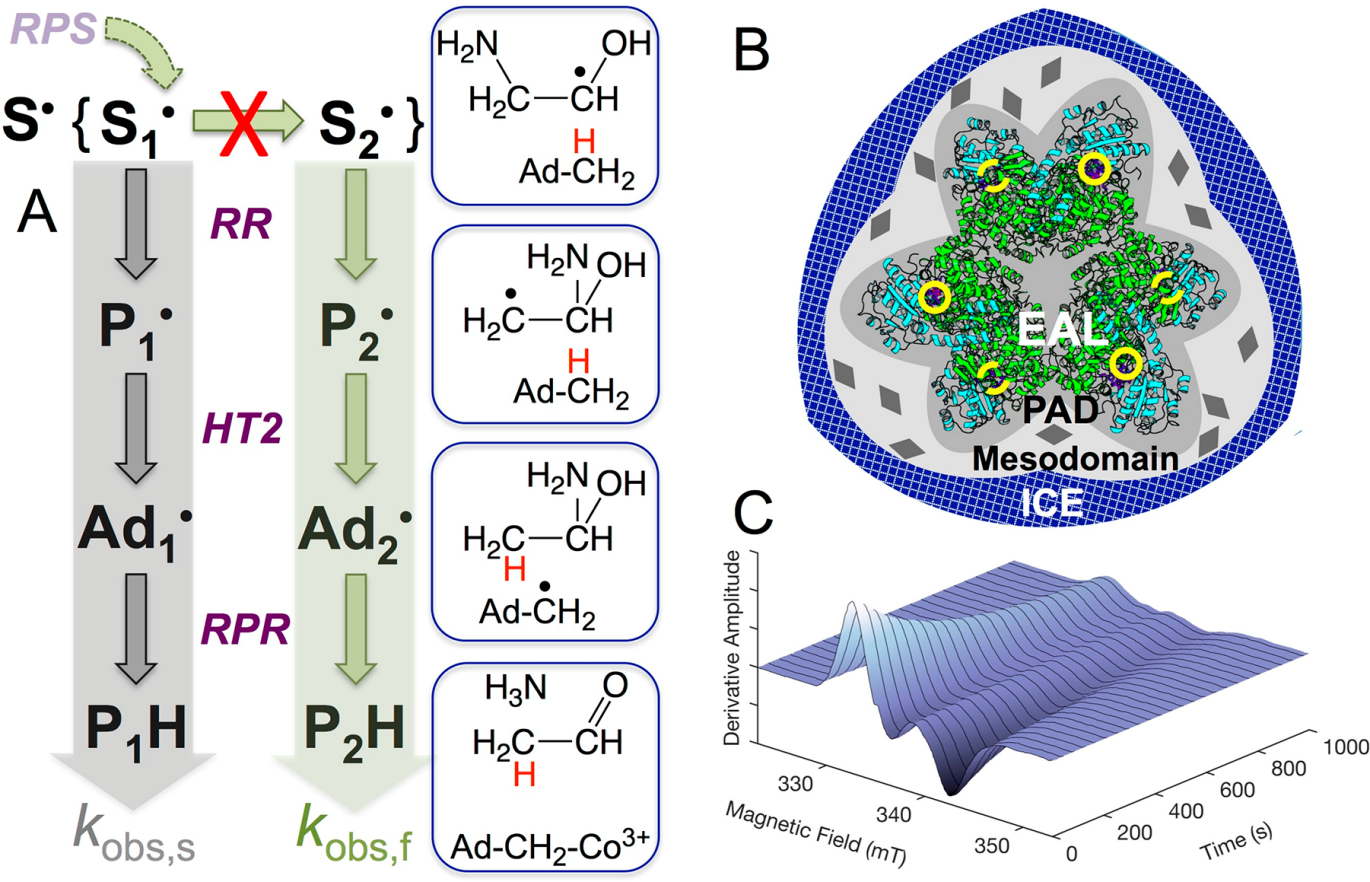
Depiction of the substrate radical decay reaction pathways and active site reactant structures in EAL, situation of the enzyme in the low-temperature, frozen aqueous solution system, and representative time-resolved decay of the substrate radical EPR spectrum. (A) Decay reaction of the substrate radical canonical state, **S**^•^, in EAL active sites cryotrapped in either the **S**_**1**_^•^ substate or the **S**_**2**_^•^ substate. Radical pair separation (RPS) forms the capture substate, **S**_**1**_^•^. Native path of **S**_**1**_^•^ conversion to **S**_**2**_^•^ is blocked (red X) at 217 K, leading to observed decay through native (green) or non-native (gray) pathways by radical rearrangement (RR) and hydrogen transfer 2 (HT2) steps, followed by radical pair recombination (RPR) to diamagnetic products. (B) Depiction of the solid ice boundary and fluid mesodomain (light gray) and protein associated domain (PAD, medium gray) phases that surround the EAL protein oligomer (active sites, yellow circles) in frozen solution containing aminoethanol (0.6% v/v) and sucrose (0–5% w/v). Solid sucrose hydrates are depicted as diamonds (dark gray). (C) Time-dependence of the substrate radical EPR spectrum following *T*-step reaction initiation.

Here, the kinetics of **S**_**1**_^•^ and **S**_**2**_^•^ decay reactions in EAL in the low-temperature aqueous-aminoethanol mesodomain system are measured in the presence of added sucrose (2, 4, 5% w/v) at 217 K. We have shown that solutions of sucrose form an interstitial mesodomain in the frozen aqueous solution,^33^ as reported by others.^34,35^ However, unlike the homogeneous mesodomains formed by cryosolvents, such as dimethyl sulfoxide, glycerol, and aminoalkanols,^28,29,36^ the aqueous-sucrose mesodomain contains a concentration-dependent fraction of solid sucrose hydrates (Figure 1B).^33^ The power-law distribution in the kinetics of **S**_**1**_^**•**^ decay in the presence of sucrose, which is not observed for the parallel decay of **S**_**2**_^•^ under the same conditions, is proposed to arise from heterogeneity in the degree of sucrose hydrate confinement communicated to each EAL active site in **S**_**1**_^•^ by solvent–protein configurational coupling. This heterogeneity in confinement is shown to resolve the S_1_^•^ substate into microstates, S_1, *i*_^•^, with each microstate, *i*, associated with a different decay rate constant, *k*_obs,s,*i*_. The results reveal that all microstates, S_1,*i*_^•^, even those distant in the configuration space from the downstream reaction-enabled state, are capable of catalysis but that the native reaction coordinate involves a collapse of the configuration space to generate the optimized reactivity of the singular **S**_**2**_^•^ substate. This refines the ensemble view of a sustained, functionally relevant, distribution of conformational states orthogonal to the reaction coordinate through all phases of the enzyme catalytic cycle.

We begin with measurements of the time-dependence of the decay of the amplitudes of the substrate radical states formed from ^1^H-aminoethanol and ^2^H-aminoethanol at added sucrose concentrations from 0 to 5% at *T* = 217 K (Figure 2 A,B; EPR spectra, Figure S1). Figure 2 plots the logarithm of the amplitude, normalized to zero-time, versus the logarithm of the decay measurement time (all logarithms are base-10). The amplitude corresponds to the difference between the low-field peak and high-field trough of the substrate radical EPR spectrum (shown in Figure 1C), measured at each time point. The log(amplitude) versus log(time) plot is convenient for distinguishing homogeneous exponential kinetics (concave-down relation) and heterogeneous distributed kinetics (linear relation). As shown previously for *T* < 220 K,^24,26^ substrate radical decay in the absence of sucrose displays biexponential kinetics, from two parallel monoexponential decays from **S**_**1**_^•^ (*k*_obs,s_) and **S**_**2**_^•^ (*k*_obs,f_). In the presence of sucrose, the decays continue to show a monoexponential fast component (concave-down curve) with a value comparable to the *k*_obs,f_ for 0% sucrose, but the slow component decay deviates from pure exponential behavior. The quasi-linear log–log plot of the slow component amplitude versus time is diagnostic of a distribution in the *k*_obs,s_ rate constant values.^19^ The power law (PL) provides convenient closed-form expressions for the rate constant distribution functions.^18^Figure 2A,B shows overlaid best fits of biexponential decay at 0% added sucrose, and the monoexponential (*k*_obs,f_) plus PL (distributed *k*_obs,s_) function (eq M2) for 2, 4, and 5% sucrose, to the time-dependence of the decay amplitudes (fitting parameters, Tables S1, S2).

**Figure 2:**
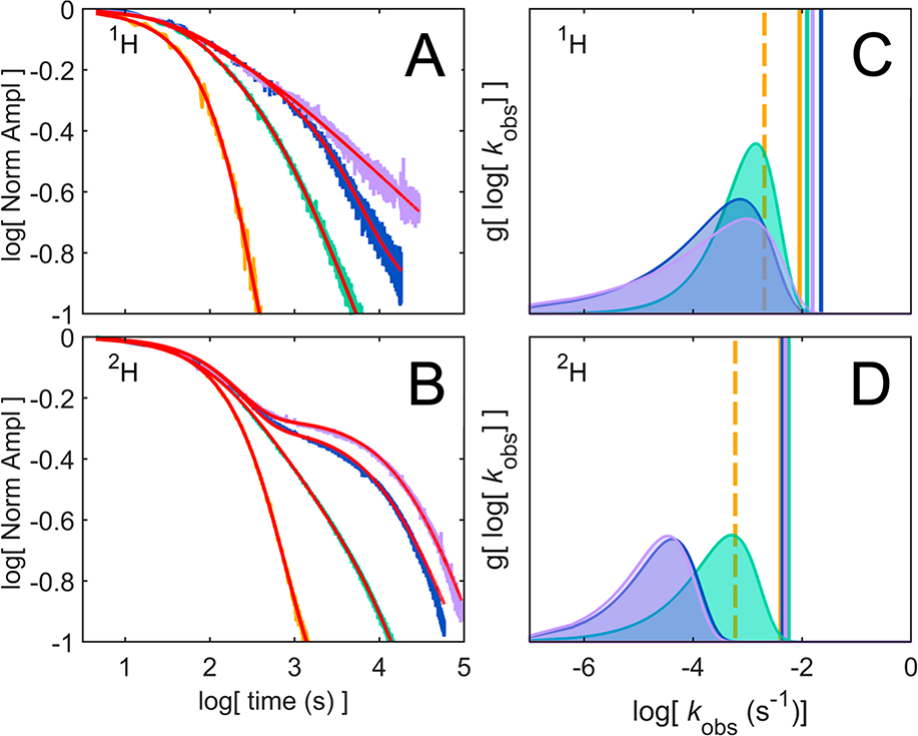
Time-dependence of the substrate radical EPR amplitude and corresponding decay component amplitudes. (A,B) Time-dependence of the EPR amplitude of the ^1^H- and ^2^H-substrate radical in the presence of 0 (orange), 2 (green), 4 (blue), and 5 (mauve) % w/v sucrose, plotted in log–log form. The normalized amplitude corresponds to the difference between the low-field peak and high-field trough of the EPR spectrum (Figure 1C) at each time point, normalized to the initial peak minus trough amplitude. Overlaid biexponential (0% sucrose) and monoexponential plus power law (2, 4, 5% sucrose) fits to the decays are shown (red). (C,D) Different components (or populations) contributing to decay time-dependence of the ^1^H- and ^2^H-substrate radical in panels A and B (color code matched), plotted as a function of the logarithm of their corresponding rate constants. Monoexponential decay components are marked by vertical sticks of arbitrary height and include fast decay components (*k*_obs,f_) under all conditions as well as the slow decay component (*k*_obs,s_) in the absence of sucrose (dashed vertical line). Relative contributions of distributed slow decay components in the presence of sucrose (*k*_obs,s,*i*_) are represented by the probability distribution function, *g*[log*k*], obtained by Laplace transformation of the power law fits to the decays in panels A and B. The distribution curves are highlighted by shading.

Figure 2 panels C and D show the fitted, sucrose concentration-independent monoexponential *k*_obs,f_, *k*_obs,s_ values at 0% sucrose (discrete values, vertical bars), and the rate constant distribution for the slow component in the presence of sucrose, *g*[log(*k*_obs,s_)]. The *g*[log(*k*_obs,s_)] distribution curves (Figure 2C,D) are obtained by Laplace transformation of the PL fits to the decays (Figure 2A,B) in the presence of sucrose.^18^ The Laplace transformation of the time domain data provides the amplitudes, or relative contributions, of the different decay components,^18,19^ and these are plotted as a function of the corresponding first-order rate constants in Figure 2C,D.^18,19^ The *g*[log(*k*_obs_)] distributions lie around the monoexponential decay *k*_obs_ values for the addition of 2% sucrose for both ^1^H- and ^2^H-substrate. The addition of sucrose to 4 and 5% causes additional broadening for ^1^H-substrate, and an *en bloc* shift of *g*[log(*k*_obs,s_)] for ^2^H-substrate to lower values. The trends of the discrete *k*_obs,f_ and mean *k*_obs,s_ values with added sucrose are shown in Figure 3A. Figure 3B shows growth in the amplitude of the slow component for both ^1^H-substrate and ^2^H-substrate with an increase in added sucrose. In terms of the general model for the decay (Figure 1A), this trend signifies an increase in **S**_**1**_^•^ population relative to **S**_**2**_^•^, which is essentially complete at 2% sucrose.

**Figure 3:**
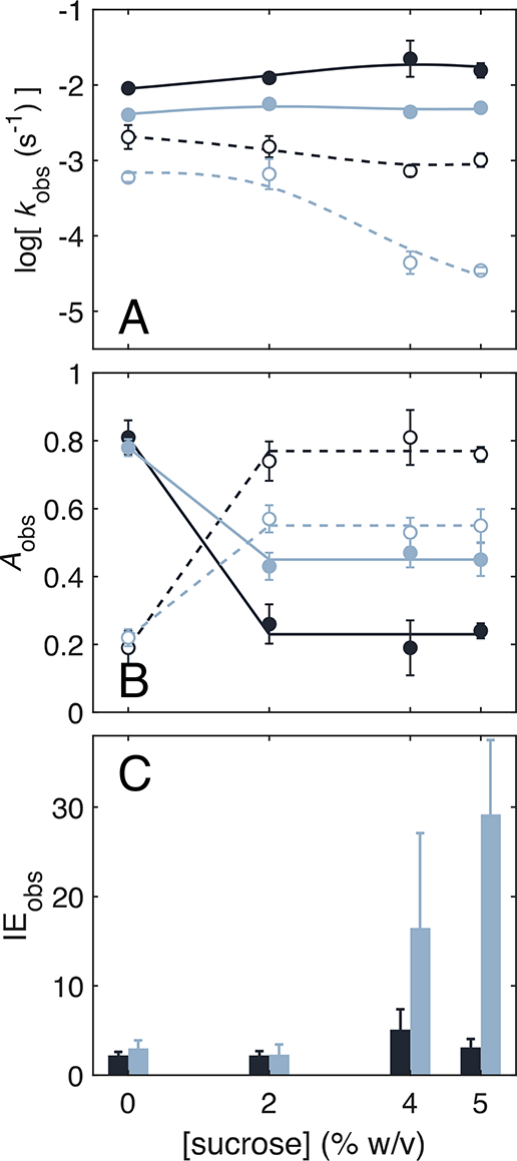
Sucrose concentration dependence of observed monoexponential and mean distributed rate constants, and corresponding amplitudes. (*A*) Monoexponential (*k*_obs,f_; *k*_obs,s_ in absence of sucrose) and mean (*k*_obs,s_ in the presence of sucrose) observed rate constants for decays of **S**_**1**_^•^ (dashed line) and **S**_**2**_^•^ (solid) for ^1^H- (black) and ^2^H- (gray) substrate. (*B*) Corresponding observed normalized weights (amplitudes) of monoexponential and distributed components. Data points and error bars represent mean and standard deviation for three separate measurements. (*C*) Histogram of observed ^1^H/^2^H isotope effects on decay of **S**_**2**_^•^ (from monoexponential *k*_obs,f_ values) and **S**_**1**_^•^ (from monoexponential *k*_obs,s_ in absence of sucrose; mean of *k*_obs,s_ distribution in the presence of sucrose).

Perturbation of the reactant and protein structure by sucrose was assessed. EPR spectra of the cob(II)alamin-substrate radical pair are sensitive to the active site environment of the substrate radical and to the separation distance of cob(II)alamin and the substrate radical within the protein. The distance between the carbon-1 radical center of the substrate radical and Co(II) in cobalamin [*r*(Co–C1^•^)] varies from 9 to >11 Å for different substrates and EAL mutants,^37^ consistent with high resolution EAL protein structures.^38^ The native cob(II)alamin-substrate radical pair line shape is maintained from 0 to 5% sucrose for both the ^1^H- and ^2^H-substrate (Figure S1). Cob(II)alamin was formed in EAL by photolysis of adenosylcobalamin bound in EAL in the presence of a bound substrate analog (Figure S2).^39^ The resolved, multifeature line shape of the magnetically isolated cob(II)alamin in EAL depends on corrin ring conformation-dependent *g*-value anisotropy, and Co(II) hyperfine and Co(II)-^14^N axial ligand superhyperfine interactions.^40^ The cob(II)alamin line shape in EAL is unchanged from 0 to 5% sucrose (Figure S2). Together, these results show that sucrose does not detectably perturb the native reactant and protein structure in and around (within ∼10 Å) the active site.

Sucrose influences the observed ^1^H/^2^H isotope effects on substrate radical decay kinetics. The observed ^1^H/^2^H isotope effect on *k*_obs,f_ (IE_obs,f_; reaction from **S**_**2**_^•^) maintains a constant value (mean, 3 ± 1) over 0 to 5% sucrose (Table S4.3). In contrast, the decrease in the mean value of *k*_obs,s_, <*k*_obs,s_>, for ^2^H-substrate relative to ^1^H-substrate leads to an approximately 10-fold increase in IE_obs,s_ over the same range of added sucrose concentrations. These results suggest that, for the distributed **S**_**1**_^•^ decay reaction, 4% and 5% sucrose have a selective effect in slowing the H-isotope-dependent HT step, relative to the RR step, leading to manifestation of an IE_obs,s_ comparable to the maximum value of ∼25 predicted for EAL (Figure 3C).^26^

Sucrose perturbs the decay kinetics of **S**_**1**_^•^, but not **S**_**2**_^•^. The decay from **S**_**2**_^•^ is monoexponential for both ^1^H- and ^2^H-substrate, and the corresponding rate constant, *k*_obs,f_, is not significantly influenced in the presence of sucrose (Figure 2). In contrast, sucrose introduces a concentration-dependent distribution in the decay kinetics for **S**_**1**_^•^, signified by a PL form for *k*_obs,s_ (Figure 2). For both ^1^H- and ^2^H-substrate at 2% sucrose, <*k*_obs,s_> is not substantially shifted from the single exponential value at 0% sucrose. At 4 and 5% sucrose, the centroid of the ^2^H-substrate distribution is significantly shifted to lower *k*_obs,s_ values, while for ^1^H-substrate, the shift in <*k*_obs,s_> is primarily due to growth of a low-frequency “tail.” The selective effects of sucrose on **S**_**1**_^•^ decay kinetics are remarkable because **S**_**1**_^•^ and **S**_**2**_^•^, cryotrapped in different EAL active sites, are exposed to the same aqueous aminoethanol-sucrose mesodomain environment. We thus seek a model in which the distributed *k*_obs,s_ values for **S**_**1**_^•^ in the presence of sucrose arise from an intrinsic property of this state, which is different from **S**_**2**_^•^.

Sucrose, as a small molecule, is unique in low-*T* mesodomain systems in its ability to induce distributed *k*_obs,s_ values. Distribution of *k*_obs.s_ is not observed for aminoethanol alone (present as substrate at 0.6% v/v),^24,26^ 2-aminopropanol (present as substrate, 0.1% v/v),^25^ or for dimethyl sulfoxide (DMSO; 2% v/v) or glycerol (2% v/v) in the presence of 0.6% aminoethanol. Sucrose, aminoalkanols, DMSO, and glycerol are all well-characterized viscosigens and eutectic solution formers in concentrated low-*T* aqueous mixtures,^41^ that also display preferential exclusion from the protein hydration layer.^31^ Thus, in the frozen solution samples of EAL, sucrose^33^ and the cryosolvents^28,29^ reside in the mesodomain. However, a distinguishing feature of concentrated, low-*T* aqueous-sucrose phases is the formation of ordered, solid sucrose-hydrate inclusions.^35,42^ The presence of a solid sucrose hydrate component in the mesodomain system in frozen solution in the absence of protein is demonstrated by the volume excluded by the hydrates.^33^ Solid sucrose hydrate structures provide interfaces capable of strong hydrogen bonding that are embedded in the glassy aqueous-sucrose mesodomain phase. Thus, sucrose hydrates in the mesodomain introduce heterogeneous confinement directly to the PAD solvent layer, owing to differences in crystallite proximity and orientations (Figure 1B). The heterogeneity in PAD confinement traps and stabilizes different solvent-coupled protein configurations from the native ensemble for times that exceed the time scale of the substrate radical decay reaction. This contrasts with the situation in the low-*T* systems frozen in the presence of the cryosolvents, in which the PAD is surrounded by a homogeneous mesodomain of nominally uniform thickness.^28,29^ Under these conditions, single-exponential decay kinetics for **S**_**1**_^•^ prevail,^24−26^ and **S**_**1**_^•^ decay kinetics remain undistributed (monoexponential) to *T* values as low as 190 K,^22^ where the estimated mesodomain viscosity is >10^5^ P.^29^ Therefore, we propose that heterogeneous sucrose hydrate confinement is the phenomenological origin of the distributed **S**_**1**_^•^ decay kinetics. Sucrose hydrate confinement stabilizes discrete configurations that represent transient native configurations along the progress coordinate through **S**_**1**_^•^ at room *T*. This is in-line with the functional coupling of the active site to protein and solvent configurations and motions,^7,11^ that are related to the transient states observed by ultrafast spectroscopies at ambient temperature,^43^ in other systems. Protein configuration changes introduced by heterogeneous sucrose confinement are subtle: The signature EPR lineshapes of the Co(II)-cobalamin cofactor and substrate radical^37^ do not evidence structural changes caused by the 2–5% sucrose on ≳1 Å length scales (Figure S1, S2). Similar distributed **S**_**1**_^•^ decay kinetics are observed when sucrose is replaced by the proteins, bovine serum albumin (BSA), or lysozyme (Figure S4). These proteins are concentrated in the low-*T* mesodomain system and introduce heterogeneous confinement to EAL, through varied orientations and proximities. The comparable *t*_0_ and *n* power law fitting parameters for 2% sucrose, BSA, and lysozyme (Table S4) show that the distribution of **S**_1,*i*_^•^ microstates is independent of the heterogeneous trapping agent and is therefore an inherent property of EAL.

The distributed decay kinetics resolves the sequence of microscopic states within the S_1_^•^ minimum that constitutes the progress coordinate. The protein structure in **S**_**1**_^•^ is configured for capture and stabilization of the substrate radical created by the radical pair separation events.^25,26^ The nascent substrate radical pair state is characterized by a large equilibrium entropy, relative to the initial bound substrate, ternary complex state (Δ*S*_0_ = 40 cal/mol/K).^44^ The large increase in entropy arises from an increase in the number of configurations in the radical pair state.^44,45^ Thus, the **S**_**1**_^•^ free energy minimum is rich in thermally accessible, microscopic configurations, **S**_1,*i*_^•^, each with probability, *P*_i_, to decay with the rate constant, *k*_obs,s,*i*_. If configurational interconversion among the **S**_1,*i*_^•^ with rate constant, *k*_int_, is more rapid than decay (*k*_int_ ≫ *k*_obs,s,*i*_), then decay occurs with a single, exchange-averaged rate constant, that lies within the envelope of the *k*_obs,s,*i*_,^20^ as observed in the absence of sucrose (Figure 2). This interpretation is supported by calculations based on a network model (Supporting Information, Text; Figure S3). However, in the presence of sucrose, the heterogeneous confinement traps the different native **S**_1,*i*_^•^ microstates within the ensemble in the *native* coupled solvent–protein configurations, leading to the condition, *k*_int_ ≪ *k*_obs,s,*i*_. This is revealed experimentally because the trapped **S**_1,*i*_^•^ cannot achieve the **S**_2_^•^ configuration and react, non-natively, to form product. Figure 4 depicts this model, by relating *k*_obs,f_ and the distribution in *k*_obs,s,*i*_ to the hierarchy of canonical macrostates (**S**^•^, **P**^•^, **PH**; see Figure 1A), substates (**S**_1_^•^, **S**_2_^•^; **P**_1_^•^, **P**_2_^•^; **PH**_1_, **PH**_2_) and detectable microstates (**S**_**1***,*i**_^•^, **P**_**1***,*i**_^•^, **PH**_**1,***i*_). At physiological temperatures, the native reaction follows a trajectory through the **S**_1_^•^ and reacts solely through the **S**_2_^•^ → **P**_2_^•^ → **PH**_2_ path (gold, Figure 4B).

**Figure 4:**
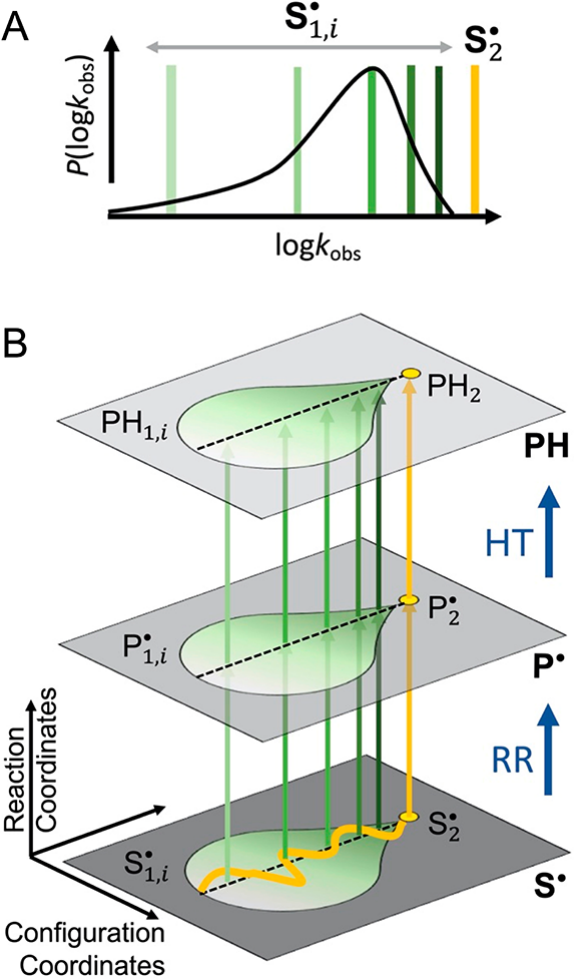
Reactivity tracking of sequential substrate radical populations and derived model for native and non-native reaction pathways. (A) Representation of the probability distribution of observed decay rate constants (corresponding to log *k*_obs,s,*i*_) under ^1^H or ^2^H conditions, through the microstates, **S**_**1,*i***_^•^, of the **S**_**1**_^•^ substate (dark to light green bars: 10% rise, 50% rise, peak, 50% fall, and 10% fall) receding from the singular native **S**_**2**_^•^ substate decay rate (gold). (*B*) Model for the non-native and native reaction pathways. Planes represent the canonical enzyme catalytic cycle states (defined by their characteristic reactant structures; see Figure 1A), substrate radical (**S**^•^), product radical (**P**^•^) and diamagnetic products (**PH**), within which reside substates (**S**_**1**_^•^, **S**_**2**_^•^; **P**_**1**_^•^, **P**_**2**_^•^; **PH**_**1**_, **PH**_**2**_) and detectable microstates (**S**_**1***,*i**_^•^, **P**_**1***,*i**_^•^, **PH**_**1,***i*_). Vertical arrows connect canonical and substate configurations through bond-making/bond-breaking chemical steps (RR, radical rearrangement; HT, hydrogen transfer), with color code match to log *k*_obs,*i*_ positions in panel A. At low *T*, non-native reaction occurs along vertical arrows from the cryotrapped **S**_**1***,*i**_^•^ without crossover. At physiological *T*, the native path is represented by the trajectory (gold) through **S**_**1***,*i**_^•^ to **S**_**2**_^•^, followed by reaction through **P**_**2**_^•^ to **PH**_**2**_.

**Scheme 1:**
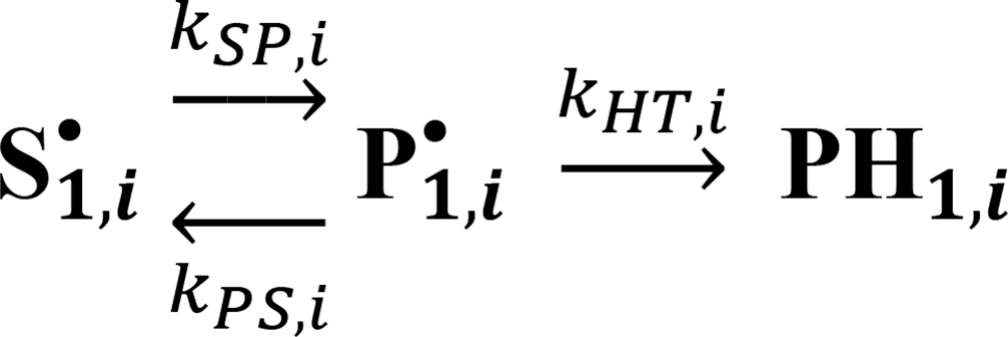
Kinetic Mechanism for Substrate Radical Decay Reaction of Each Microstate, **S**_1,*i*_^•^

The model for the distributed decay kinetics of the S_1_^•^ microstate ensemble in Figure 4 is quantified by characterizing the observed decay reaction of the substrate radical, from each microstate, **S**_1,*i*_^•^, by using the kinetic mechanism in Scheme 1. This mechanism was used previously to describe the homogeneous decay kinetics of the **S**_**1**_^•^ and **S**_2_^•^ substates for ^1^H and ^2^H substrate in the absence of sucrose.^26^ The expression for *k*_obs,s,*i*_ for the substrate hydrogen isotope conditions, ^*n*^H (*n* = 1, 2), is derived under the steady-state assumption for **P**_1,*i*_^•^, [**P**_1,*i*_^•^] ≪ [**S**_1,*i*_^•^]:^26,44,46^
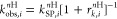
1where *k*_SP, *i*_^*n*H^ is the forward rate constant for RR, and  is the ratio of the reverse RR and HT rate constants.^26^ As for the homogeneous kinetics in the absence of sucrose, the forward and reverse rate constants for RR are considered H-isotope independent (*k*_SP,*i*_^1H^ = *k*_SP,*i*_^2H^ and *k*_PS,*i*_^1H^ = *k*_PS,*i*_^2H^), and thus, *k*_obs,s,*i*_ for ^1^H is solely rate-determined by the RR step (*k*_obs,*i*_^1H^ = *k*_SP,*i*_^1H^, or *r*_*k*,*i*_^1H^ ≪ 1).^26^ Therefore, any difference between *k*_obs,*i*_^1H^ and *k*_obs,*i*_^2H^ is attributed to *r*_*k*,*i*_^2H^. Values of the microscopic rate constants are calculated for individual *k*_obs,s,*i*_ at selected positions in the distributions for ^1^H and ^2^H substrates (Figure 4A), which correspond to sequential positions along the progress coordinate (values, Table S5). For the 2% sucrose condition, incorporation of ^2^H slows the HT step relative to reverse RR, owing to the substantial intrinsic kinetic isotope effect [predicted value for EAL,^25,26^ comparable to other B_12_ enzymes^47^]. This leads to values of *r*_*k*,*i*_^2H^ that vary from 0.82 to 4.8 from the 10% rise to 10% fall amplitudes of the distribution. Therefore, *k*_HT,*i*_^2H^ makes a relatively constant contribution to the partial rate-determination of the substrate radical decay, with an effect that varies by only 6-fold over the 800-fold change of *k*_obs, *i*_^2H^ between 10% rise and fall amplitudes. This suggests that, under the 2% sucrose condition, *k*_HT,*i*_^*n*H^ and *k*_PS,*i*_^*n*H^ are affected equivalently and, therefore, the activation free energy barriers for RR and HT are raised in concert, as the **S**_**1***,*i**_^•^ microstates recede from the native configuration identified with **S**_2_^•^ (Figure 4A). Each approximately isenthalpic **S**_**1***,*i**_^•^ microstate in the distribution follows a unique channel of non-native reaction through the configurational substate tiers (Figure 4B). The **S**_**1***,*i**_^•^ microstates that are more structurally distant from the native enabled RR configuration experience a greater energy increase upon distortion through the series of configurational states necessary to achieve the transition to the corresponding **P**_**1***,*i**_^•^ microstate in Figure 4B. This effect is replicated for the HT reaction of each **P**_**1***,*i**_^•^ to form **PH**_**1,*i***_, in line with the tandem change in RR and HT barriers.

The 4% sucrose condition introduces a shift in the log *k*_obs,*i*_^2H^ distribution to lower values by approximately 1 order of magnitude (Figure 2D). The shift is uniform, as shown by the comparable *t*_0_ and *n* parameters for the power law fits of decay curves for 2, 4, and 5% sucrose (Table S2). This could, in principle, arise from individual or conjugate effects involving all of the microscopic rate constants in eq 1. Limiting assumptions of an effect on only *k*_HT,*i*_ or *k*_SP,*i*_ leads to comparable increases in *r*_*k*,*i*_^2H^ for 4% versus 2% sucrose (Table S6). A dominant effect on *k*_HT,*i*_ is favored because it produces the observed pronounced “tail” in the *k*_obs,*i*_^1H^ distribution, rather than the *en bloc* shift shown by the *k*_obs,*i*_^2H^ distribution (Figure 2C,D), and it is consistent with the approach of the mean IE_obs,s_ for 4, 5% sucrose to the predicted intrinsic IE (Figure 3C, Table S3). The presence of sucrose crystallites in the mesophase at higher added sucrose^33^ would increase confinement of each microstate in the native distribution, which could influence the protein-internal encounter-dependent HT more than RR.

Overall, our approach provides fundamental insight into how an enzyme conducts strongly adiabatic chemical steps. Reactivity tracking reveals that each parsed **S**_**1,*i***_^•^ microstate, from “capture” toward “enabled” configuration, is capable of conducting the core sequence through both RR and HT (Figure 4B), with significant catalysis of ≳0.1% for *k*_SP_ relative to the native, optimized value for **S**_**2**_^•^. Thus, the colocation of active site elements and their connections through the protein to solvent afford a significant degree of catalysis. The principal conclusion is that there is a pronounced narrowing of the reactivity-detected configuration space in the lead-up to and execution of the native core sequence of covalent chemical events. The substrate radical substate, **S**_**2**_^•^, which is enabled for the native adiabatic RR and subsequent HT reactions, is detectably singular, relative to the distribution of precursor configurations that compose the capture substate, **S**_**1**_^•^. This refines the paradigm, that the progress coordinate through all phases of the catalytic cycle, from substrate binding to product release, and all levels of the dynamical hierarchy, corresponds to a comparable, relatively broad distribution of conformational states.^2,3,7^ Under native physiological temperature conditions, the nascent substrate radical capture configuration (left-hand side, Figure 1A, Figure 4B) is reconfigured, on a time-scale shorter than the ensuing chemical steps, into the enabled configuration (right-hand side, Figure 1A, Figure 4B), which then conducts the RR and HT reactions. The full set of native sequential configurational changes are required, both for optimization of the catalysis, and for fidelity in handling the highly reactive radical species.^48^ The results and model also reveal a molecular mechanical basis for the influence of solvent–protein-reaction configurational coupling on enzyme function under crowding confinement in vivo and suggest the existence of a latent, expanded target set for effector (drug) modulation of activity.

## Supporting Information Available

Materials and methods, supporting text (multistate kinetics model), figures showing EPR spectra of the cob(II)alamin-substrate radical pair and cob(II)alamin in EAL, representative multistate model, and observed rate constants under protein crowding, tables showing kinetic parameters for biexponential and power law function fits to observed ^1^H- and ^2^H-substrate radical decays at different added sucrose concentrations, sucrose concentration dependence of ^1^H/^2^H isotope effect on observed rate constants, kinetic parameters for biexponential and power law function fits to observed substrate radical decays under protein crowding, observed and microscopic rate constants for decay reaction of substrate radical microstates across S_1, *i*_^•^ microstate distributions under ^1^H-substrate and ^2^H-substrate conditions (2% sucrose), and observed and microscopic rate constants for decay reaction of substrate radical microstates across S_1,*i*_^•^ microstate distributions under ^1^H-substrate and ^2^H-substrate conditions (4% sucrose), and supporting references (PDF)
