# Supplementary material for: Reactivity Tracking of an Enzyme Progress Coordinate
Source: J Phys Chem Lett. 2023 Aug 4;14(32):7157–64. doi: 10.1021/acs.jpclett.3c01464 (PMC10440813; doi:10.1021/acs.jpclett.3c01464)
Supplement: Supplementary file 1 — jz3c01464_si_001.pdf [file jz3c01464_si_001.pdf]

Supporting Information for

Reactivity Tracking of an Enzyme Progress Coordinate

Wei Li,[a] Meghan Kohne,[a] and Kurt Warncke[a,]*

[a]*Department of Physics, Emory University, Atlanta, GA 30322*

Corresponding Author

* Kurt Warncke

Email: kwarncke@physics.emory.edu

This PDF file includes:

Page

Materials and Methods S3

Enzyme and EPR sample preparation

Time-Resolved EPR Measurement

Fitting of substrate radical decay kinetics

Supporting Text S6

Multi-state kinetics and calculation of mean observed decay rate constants

Figures S10

Figure S1. EPR spectra of the cob(II)alamin-substrate radical pair in EAL.

Figure S2. Cob(II)alamin in EAL under anaerobic condition with 0, 2, 4, and 5 % (w/v) sucrose.

Figure S3. Representative multi-microstate model and dependence of the observed decay rate constants and amplitudes on microstate interconversion rate.

Figure S4. Observed first-order rate constants and power law distribution of rate constants for different components of the ^{1}H-substrate radical decay in EAL samples cryotrapped in the presence of the proteins, bovine serum albumin and lysozyme.

Tables S14

Table S1. Kinetic parameters for biexponential and power law function fits to the observed ^{1}H-substrate radical decay reactions at 0, 2, 4, and 5 % sucrose.

Table S2. Kinetic parameters for biexponential and power law function fits to the observed ^{2}H-substrate radical decay reactions at 0, 2, 4, and 5 % sucrose.

Table S3. Sucrose concentration dependence of the $^{1}H/^{2}H$ isotope effect on the observed substrate radical decay rate constants.

Table S4. Kinetic parameters for biexponential and power law function fits to the observed ^{1}H-substrate radical decay reactions in the presence of bovine serum albumin (BSA) and lysozyme (LYZ), and for 2% sucrose control.

Table S5. Observed rate constant, and microscopic rate constants and rate constant ratio parameter, for decay reaction of substrate radical microstates across $S^{\bullet}_{1,i}$ microstate distributions under ^{1}H-substrate and ^{2}H-substrate conditions with added 2% sucrose.

Table S6. Observed rate constant, and microscopic rate constants and rate constant ratio parameter, for decay reaction of substrate radical microstates across the $S^{\bullet}_{1,i}$ microstate distributions under ^{1}H and ^{2}H conditions for two limiting cases of the effect of 4% sucrose on the microscopic rate constants, relative to 2% sucrose.

SI References S18

Materials and Methods

Enzyme and EPR sample preparation. EAL was obtained from the *Escherichia coli* overexpression system that incorporates the cloned *S. typhimurium* EAL coding sequence,[1] and purified with modifications, as described.[2] EPR samples contained 20 μM EAL (120 μM active sites), 480 μM coenzyme B_{12} (adenosylcobalamin, AdoCbl), 100 mM aminoethanol [natural isotope abundance or ^{2}H-labeled, 1,1,2,2-^{2}H-aminoethanol (Cambridge Isotope Laboratories, Inc., Tewksbury, MA)], and 10 mM potassium phosphate buffer (pH 7.5) in a final volume of 300 μL, at pH 7.5. Added sucrose concentrations of 0-5 % weight/volume (w/v) were achieved by using USDA Brix conversion tables.[2] Procedures for cryotrapping of the substrate radical have been described in detail.[3]

Time-Resolved EPR Measurement. EPR spectra were collected at 217 K by using a Bruker E500 ElexSys EPR spectrometer equipped with a Bruker ER4123 SHQE cavity. The full-spectrum, time-resolved EPR spectroscopy methods have been described in detail.[3] Briefly, EPR samples were held at a staging temperature of 160−180 K in the ER4131VT cryostat system in the spectrometer, and temperature was step-increased to the decay measurement value of 217 K. The time from initiation of the temperature step to the start of acquisition of the first spectrum was 30−60 s. Repetitive acquisition of EPR spectra (24 s sweep time; 2.56 ms time constant) proceeded for the duration of the decay. The temperature at the sample was determined by using an Oxford Instruments ITC503 temperature controller with a calibrated model 19180 4-wire RTD probe, which has ±0.3 K accuracy. The temperature gradient over the height (long-axis) of the upright sample in the EPR cavity was measured to be ±0.2 K by using an ultrathin, T-band thermocouple

probe.[3] The ER4131VT cryostat/controller system thus provided a temperature stability of approximately ±0.4 K over the length of the EPR sample in the cavity.

Fitting of substrate radical decay kinetics. All data processing programs were coded in Matlab (Mathworks, Natick, MA). The time-dependence of the observed peak-to-trough amplitude of the substrate radical EPR spectrum was fitted to the biexponential function (Eq. M1; 0% sucrose; $A_{obs,s}$, $A_{obs,f}$, normalized component amplitudes; $k_{\text{obs,s}}$, $k_{\text{obs,f}}$, first-order rate constants) or monoexponential plus power law functions (Eq. M2; 2-5% sucrose; $k_{obs,f}$, n, t_0 are adjustable parameters):

$$A_{\text{obs}}(t) = A_{\text{obs,f}}\exp\left(-k_{\text{obs,f}}t\right) + A_{\text{obs,s}}\exp\left(-k_{\text{obs,s}}t\right) \qquad \text{Eq. M1}$$

$$A_{\text{obs}}(t) = A_{\text{obs,f}}\exp\left(-k_{\text{obs,f}}t\right) + A_{\text{obs,s}}(1 + t/t_0)^{-n} \qquad \text{Eq. M2}$$

The power law dependence implies a distribution of monoexponential rate constants, $k_{\text{obs,s,i}}$, for decay from states, *i*.[4] The probability, P_{i}, of state *i* with associated decay rate constant, $k_{\text{obs,s,i}}$, is given by:

$$P\left(k_{\text{obs,s,i}}\right) = \frac{k_{\text{obs,s,i}}^{n-1}\exp\left(-k_{\text{obs,s,i}}t_0\right)}{\Gamma(n)}t_0^n \qquad \text{Eq. M3}$$

where Γ(*n*) is the gamma function. The mean value of $k_{obs,s,i}$ is given by:

$$< k_{\text{obs,s,i}} >= n/t_0 \qquad \text{Eq. M4}$$

For broad distributions in k_i, the following logarithmic expression is convenient for plotting the probability:

$$P(\log[k_{\mathrm{obs,s,i}}]) = \ln 10 \times P(k_{\mathrm{obs,s,i}}) \times k_{\mathrm{obs,s,i}} \qquad \text{Eq. M5}$$

Supporting Text

Multi-state kinetics and calculation of mean observed decay rate constants.

In a system with finite number of connected substates, in which each substate has a potential decay pathway, the time dependence of the concentration of each intermediate, $\mathrm{C_i}$, is described as

$$\frac{d[\mathrm{C_i}]}{dt} = \sum_{\mathrm{j}} \left(k_{\mathrm{ji}}[\mathrm{C_j}] - k_{\mathrm{ij}}[\mathrm{C_i}]\right) - k_{\mathrm{i}}[\mathrm{C_i}] \qquad \mathrm{Eq.\,S1}$$

where k_{ij} and k_{ji} are the rate constants for interconversion between intermediates i and j, and k_{i} is the intrinsic rate constant characterizing the decay reaction from intermediate i. Eq. S1 can be rewritten as

$$\hat{T}\vec{c} = \frac{d\vec{c}}{dt} \qquad \mathrm{Eq.\,S2}$$

in which $\vec{c}$ is a column vector that represents intermediate concentrations, as a function of time, and the transition matrix $\hat{T}$ is defined as

$$\hat{T} = \begin{bmatrix} -\sum_{\mathrm{j}} k_{\mathrm{1j}} - k_1 & k_{21} & \cdots & \cdots & k_{\mathrm{n1}} \\ k_{12} & -\sum_{\mathrm{j}} k_{\mathrm{2j}} - k_2 & & & \\ \vdots & & \ddots & & \vdots \\ \vdots & & & \ddots & \\ k_{\mathrm{1n}} & \cdots & \cdots & & -\sum_{\mathrm{j}} k_{\mathrm{nj}} - k_{\mathrm{n}} \end{bmatrix} \qquad \mathrm{Eq.\,S3}$$

Since the cryo-trapped substrate radical in the $\mathbf{S}^{\bullet}$ state proceeds into product without replenishment between 190 K and 250 K [3], the system is in a non-steady state, and the observed total population decay ($\vec{c}_{\mathrm{obs}}$) can be written as a linear combination of time-independent eigenvectors $\vec{e}_{\mathrm{i}}$ and their time-evolving part $\exp[-\lambda_{\mathrm{i}}t]$.

$$\vec{c}_{\mathrm{obs}}(t) = \sum \alpha_{\mathrm{i}}\vec{e}_{\mathrm{i}}\exp[-\lambda_{\mathrm{i}}t] \qquad \mathrm{Eq.\,S4}$$

$$\hat{T}\left[\vec{e}_{\mathrm{i}}\,\exp[-\lambda_{\mathrm{i}}t]\right] = -\lambda_{\mathrm{i}}\vec{e}_{\mathrm{i}}\,\exp[-\lambda_{\mathrm{i}}t] \qquad \mathrm{Eq.\,S5}$$

The $-\lambda_{\mathrm{i}}$ are the eigenvalues of transition matrix $\hat{T}$. Because these intermediates are degenerate substates along the reaction coordinate, all the eigenvalues are assumed to be unique. The α_i are the linear coefficients, which are explicitly obtained from the expression for the initial conditions,

$$\vec{c}_{\mathrm{obs}}(0) = \sum \alpha_{\mathrm{i}}\vec{e}_{\mathrm{i}} \qquad \mathrm{Eq.\,S6}$$

Furthermore, the experimental signal resolved from EPR spectroscopy is the total amplitude from all substrate radical intermediates:

$$A_{\mathrm{tot}}(t) = \mathrm{sum}(\vec{c}_{\mathrm{obs}}) = \sum \alpha_{\mathrm{i}}\,\mathrm{sum}(\vec{e}_{\mathrm{i}})\,\exp[-\lambda_{\mathrm{i}}t] \qquad \mathrm{Eq.\,S7}$$

The observation of experimental power-law decay of total amplitude $A_{\mathrm{tot}}(t)$, therefore represents a linear combination of eigen-phases (Eq. S7), with the observed amplitude $A_{\mathrm{obs,i}} = \alpha_{\mathrm{i}}\,\mathrm{sum}(\vec{e}_{\mathrm{i}})$ and the observed rate constants $k_{\mathrm{obs,i}} = \lambda_{\mathrm{i}}$. Namely,

$$A_{\mathrm{tot}}(t) = \sum A_{\mathrm{obs,i}} \exp\left(-k_{\mathrm{obs,i}} t\right) \qquad \mathrm{Eq.\,S8}$$

To build a simulation with *n* potential intermediates, a $n \times n$ transition matrix $\hat{T}$ is generated. Here we assume that the interconversion rate constants are uniform,

$$k_{\mathrm{ij}} = k_{\mathrm{ji}} = \nu \qquad \mathrm{Eq.\,S9}$$

Eq. S9 is an approximation, that is helpful to simplify the model and to better illustrate the behavior of the system. The influence of the relative values of microstate interconversion rates and decay rates on the observed decay rate constants and amplitude is demonstrated by using a simplified, four-state model (*n* = 4) (Figure S3A). This model represents all possible interconversion pathways, k_{ij}, k_{ji}, which can be tailored to represent the degree of local or global coupling of the microstates. Each microstate can decay with rate constant, k_{i} $(\mathrm{i} = 1-4)$. The total population decay can be described as a linear combination of exponential functions [Eq. S8].

Figure S3 B, C show simulations of the dependence of $k_{\mathrm{obs,i}}$ and $A_{\mathrm{obs,i}}$ on the effective interconversion rate ν $\left(= k_{\mathrm{ij}} = k_{\mathrm{ji}}\right)$, assuming decay rate constants of k_1, k_2, k_3 and k_4 of $10^{-3}, 10^{-5}, 10^{-6}$ and 10^{-7} s^{-1}, respectively, with an initial normalized population of 0.25 for each microstate. When $\nu < 10^{-8}$ s^{-1}, indicating that the interconversion rates are

significantly slower (≤10-fold) than the decay rates, the total population decay can be described as the simple addition of each substate decay. This corresponds to the PL distribution observed for the decay of EAL for $k_{obs,s}$ in the presence of sucrose (Figure 2 C, D), where interconversion of the configurational microstates within $\mathbf{S_1^{\bullet}}$ is blocked on the time scale of decay by the sucrose confinement effect. When $10^{-8} < \nu < 10^{-3}$ s^{-1}, with increasing ν, all observed rates become faster, and the spread between these rates narrows. When $\nu > 10^{-3}$ s^{-1}, the interconversion rate ν is more rapid than decay rates, and only one averaged exponential decay is observed, $\langle k_{\mathrm{i}} \rangle$, that lies within the envelope of the k_{i}. This is the condition for substrate radical decay from the $\mathbf{S_1^{\bullet}}$ state in EAL in the absence of sucrose (0% condition), for which the decay is monoexponential (single rate constant, $k_{\mathrm{obs},s}$) (Figure 2 C, D).

The simple multi-state kinetic model considered here supports the interpretation of the monoexponential and power-law decay kinetics of the $\mathbf{S_1^{\bullet}}$ state in EAL as caused by the limits of restricted interconversion (2-5 % sucrose) and rapid interconversion (0% sucrose).

Figures

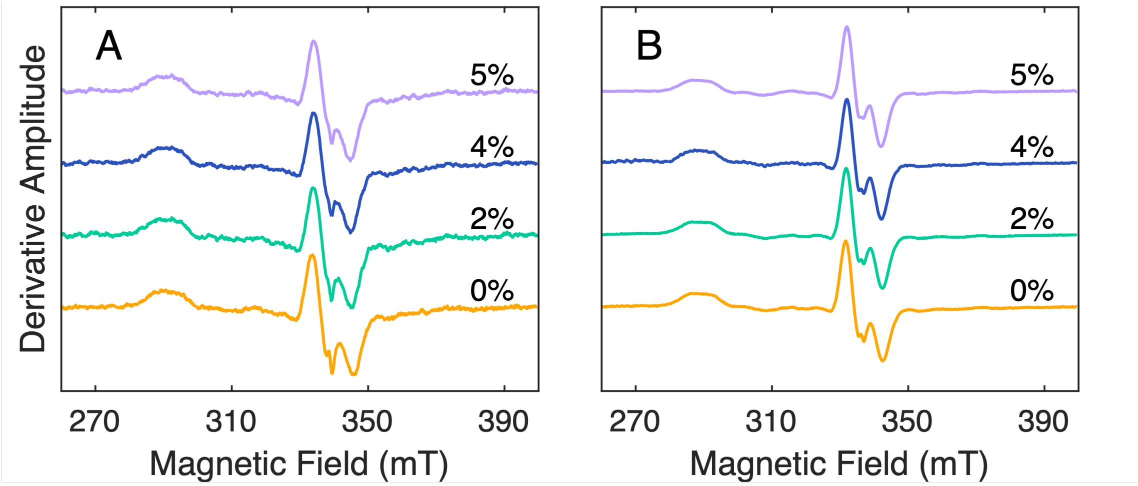

Figure S1. EPR spectra of the cob(II)alamin-substrate radical pair in EAL. (A) Radical generated by using ^{1}H-aminoethanol and (B) Radical generated by using ^{2}H-aminoethanol. Spectra were obtained in the presence of no sucrose (0%, orange), and added 2 (green), 4 (blue) and 5 (mauve) % (w/v) sucrose. EPR conditions: microwave frequency, 9.4533 GHz; microwave power, 20.5 mW; magnetic field modulation, 1.0 mT; modulation frequency, 100 kHz; temperature, 120 K; 10-scan average.

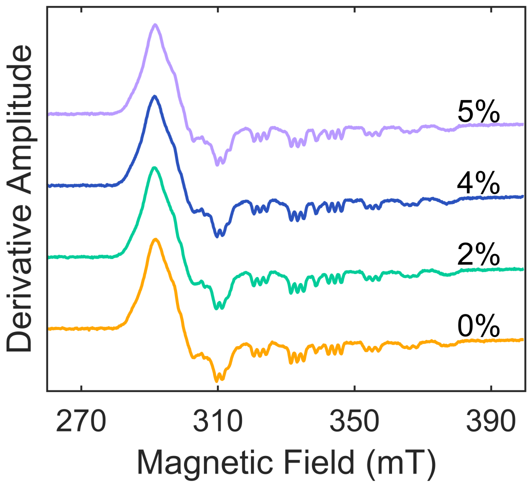

Figure S2. Cob(II)alamin in EAL under anaerobic condition with 0, 2, 4, and 5 % (w/v) sucrose. Spectra were obtained in the presence of 0 (orange), 2 (green), 4 (blue), and 5 (mauve) % w/v sucrose. EPR conditions: microwave frequency, 9.5206 GHz; microwave power, 2.0 mW; magnetic field modulation, 1.0 mT; modulation frequency, 100 kHz; temperature, 120 K; 8-scan average.

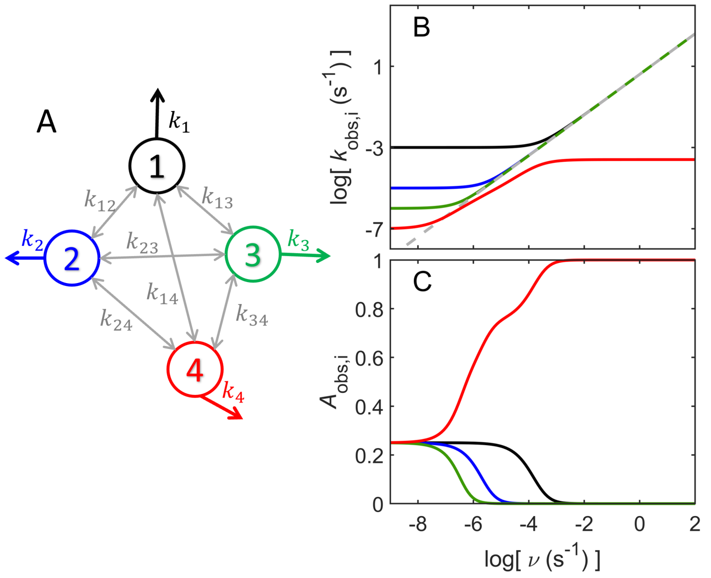

Figure S3. Representative multi-microstate model and dependence of the observed decay rate constants and amplitudes on microstate interconversion rate. (A) Network of microstates, *i* (circles), interconversion pathways (double-end arrows; rate constants, k_{ij}) and decay pathways (single-end arrows; rate constants, k_{i}) for multi-microstate system with *n*=4. (B) Observed rate constants. It is assumed that $k_{\mathrm{ij}} = k_{\mathrm{ji}} = \mathrm{\nu}$, for simplicity. The initial normalized population of each microstate is 0.25. Gray dashed line in shows $n \times \mathrm{\nu}$. (C) Observed normalized amplitudes of the system. The decay rate constants are: k_1=10^{-3} s^{-1} (black), k_2=10^{-5} s^{-1} (blue), k_3=10^{-6} s^{-1} (green), and k_4=10^{-7} s^{-1} (red).

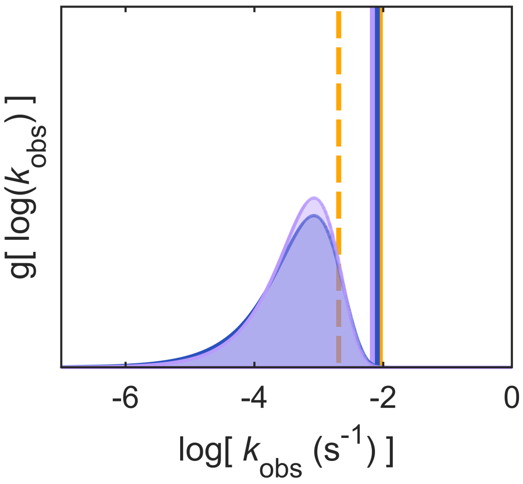

Figure S4. Observed first-order rate constants and power law distribution of rate constants for different components of the [1]H-substrate radical decay in EAL samples cryotrapped in the presence of the proteins, bovine serum albumin (BSA) and lysozyme, compared to control, EAL-only sample. The control decay was fitted by a bi-exponential function, and the obtained $k_{obs,f}$ and $k_{obs,s}$ values are shown by solid and dashed sticks, respectively (yellow). BSA (3.0 mM, mauve) or lysozyme (3.2 mM, blue) was added to mimic the heterogeneous environment created by the sucrose hydrates in the frozen solution mesodomain system. The signal decay curves for the samples in the presence of BSA and lysozyme were fitted by the single exponential +power law function. The $k_{obs,f}$ values for the exponential fast decay component, are marked on the ordinate by sticks of arbitrary height. The distribution of $k_{obs,s}$ values are depicted by solid envelopes. One trial was measured for each condition. Fitting parameters are listed in Table S4.

Tables

Table S1. Kinetic parameters for biexponential and power law function fits to the observed [1]H-substrate radical decay reactions at 0, 2, 4, and 5 % sucrose.

[sucrose] (w/v %)	$A_{obs,f}$	$k_{obs,f}$ (s^{-1})	$A_{obs,s}$	t_0 (s)	n	$\langle k_{obs,s} \rangle$ (s^{-1})	R^2
0	0.81 ± 0.05	$9.0(\pm 0.9) \times 10^{-3}$	0.19 ± 0.05			$2.0(\pm 0.7) \times 10^{-3}$	0.9957
2	0.26 ± 0.06	$1.2(\pm 0.2) \times 10^{-2}$	0.74 ± 0.06	$5.8(\pm 2.0) \times 10^{2}$	0.82 ± 0.12	$1.5(\pm 0.5) \times 10^{-3}$	0.9944
4	0.19 ± 0.08	$2.2(\pm 1.2) \times 10^{-2}$	0.81 ± 0.08	$4.9(\pm 2.3) \times 10^{2}$	0.36 ± 0.17	$7.2(\pm 1.2) \times 10^{-4}$	0.9808
5	0.24 ± 0.02	$1.6(\pm 0.3) \times 10^{-2}$	0.76 ± 0.02	$3.0(\pm 0.6) \times 10^{2}$	0.29 ± 0.02	$1.0(\pm 0.2) \times 10^{-3}$	0.9888

Table S2. Kinetic parameters for biexponential and power law function fits to the observed [2]H-substrate radical decay reactions at 0, 2, 4, and 5 % sucrose.

[sucrose] (w/v %)	$A_{obs,f}$	$k_{obs,f}$ (s^{-1})	$A_{obs,s}$	t_0 (s)	n	$\langle k_{obs,s} \rangle$ (s^{-1})	R^2
0	0.78 ± 0.03	$4.0(\pm 0.4) \times 10^{-3}$	0.22 ± 0.03			$6.0(\pm 0.8) \times 10^{-4}$	0.9997
2	0.43 ± 0.04	$5.6(\pm 0.5) \times 10^{-3}$	0.57 ± 0.04	$1.1(\pm 0.8) \times 10^{3}$	0.58 ± 0.09	$6.6(\pm 3.0) \times 10^{-4}$	0.9990
4	0.47 ± 0.04	$4.4(\pm 0.4) \times 10^{-3}$	0.53 ± 0.04	$1.4(\pm 0.3) \times 10^{4}$	0.60 ± 0.17	$4.4(\pm 1.5) \times 10^{-5}$	0.9970
5	0.45 ± 0.05	$5.0(\pm 0.5) \times 10^{-3}$	0.55 ± 0.05	$1.7(\pm 0.9) \times 10^{4}$	0.59 ± 0.28	$3.5(\pm 0.3) \times 10^{-5}$	0.9980

Table S3. Sucrose concentration dependence of the $^{1}H/^{2}H$ isotope effect on the observed substrate radical decay rate constants.†

[sucrose] (w/v%)	0	2	4	5
$IE_{obs,f}$	2.2±0.4	2.2±0.5	5.1±2.6	3.1±1.0
$IE_{obs,s}$	3.4±0.9	2.3 ± 1.1	17±11	29±8

† The $IE_{obs,s}$ values are obtained by using the mean $k_{obs,s}$ values of the PL distribution for each condition. The $IE_{obs,f}$ values are obtained by using the monoexponential decay rate constants for each condition.

Table S4. Kinetic parameters for biexponential and power law function fits to the observed ^{1}H-substrate radical decay reactions in the presence of 3.0 mM BSA and 3.2 mM lysozyme (LYZ), and for 2% sucrose (SUC) control.

crowder	$A_{obs,f}$	$k_{obs,f}\ (s^{-1})$	$A_{obs,s}$	$t_0\ (s)$	n	$\langle k_{obs,s}\rangle\ (s^{-1})$	R^2
**None**	0.81 ± 0.05	$9.0(\pm 0.9) \times 10^{-3}$	0.19 ± 0.05	—	—	$2.0(\pm 0.7) \times 10^{-3}$	0.9957
**2% SUC**	0.26 ± 0.06	$1.2(\pm 0.2) \times 10^{-2}$	0.74 ± 0.06	$5.8(\pm 2.0) \times 10^{2}$	0.82 ± 0.12	$1.5(\pm 0.5) \times 10^{-3}$	0.9944
**BSA**	0.41	6.8×10^{-3}	0.59	1.1×10^{3}	0.89	8.4×10^{-4}	0.9905
**LYZ**	0.42	7.9×10^{-3}	0.58	9.1×10^{2}	0.77	8.4×10^{-4}	0.9987

Table S5. Observed rate constant, and microscopic rate constants and rate constant ratio parameter, for decay reaction of substrate radical microstates across $\mathbf{S}^{\bullet}_{1,i}$ microstate distributions under ^{1}H-substrate and ^{2}H-substrate conditions with added 2% sucrose.

**^{1}H**	**2% sucrose**		
i	$k^{1H}_{obs,i}$ (s^{-1})	$k^{1H}_{SP,i}$ (s^{-1})*	$r^{1H}_{k,i}$ **
10%, ↑	7.24×10^{-3}	7.24×10^{-3}	0
50%, ↑	3.99×10^{-3}	3.99×10^{-3}	0
peak	1.39×10^{-3}	1.39×10^{-3}	0
50%, ↓	2.70×10^{-4}	2.70×10^{-4}	0
10%, ↓	2.89×10^{-5}	2.89×10^{-5}	0

**^{2}H**	**2% sucrose**		
i	$k^{2H}_{obs,i}$ (s^{-1})	$k^{2H}_{SP,i}$ (s^{-1})†	$r^{2H}_{k,i}$
10%, ↑	3.99×10^{-3}	7.24×10^{-3}	0.82
50%, ↑	1.62×10^{-3}	3.99×10^{-3}	1.5
peak	5.24×10^{-4}	1.39×10^{-3}	1.7
50%, ↓	5.62×10^{-5}	2.70×10^{-4}	3.8
10%, ↓	5.01×10^{-6}	2.89×10^{-5}	4.8

* In the absence of the H-isotope effect, $k^{1H}_{HT,i}$ >> $k^{1H}_{SP,i}, k^{1H}_{PS,i}$, and $k^{1H}_{SP,i}$ is rate-determining for the substrate radical decay reaction. Therefore, $k^{1H}_{obs,i}$ = $k^{1H}_{SP,i}$ [5].

** The condition, $k^{1H}_{obs,i}$ = $k^{1H}_{SP,i}$, entails $r^{1H}_{k,i}$<<1, and therefore, negligible.

† The ^{1}H/^{2}H-isotope effect on the RR step is assumed to be negligible[5]. Under this assumption, the values of $k^{1H}_{SP,i}$ and $k^{2H}_{SP,i}$ are equal, and the measured value of $k^{1H}_{SP,i}$ is used for $k^{2H}_{SP,i}$.

Table S6. Observed rate constant, and microscopic rate constants and rate constant ratio parameter, for decay reaction of substrate radical microstates across the $\mathbf{S}^{\bullet}_{\mathbf{1},i}$ microstate distributions under ^{1}H and ^{2}H conditions for two limiting cases of the effect of 4% sucrose on the microscopic rate constants, relative to 2% sucrose: Effect of 4% relative to 2% sucrose on (1) the barrier for HT, or k_{HT}, only, and (2) on the barrier for forward RR, or k_{SP}, only. The ^{2}H condition is assumed to influence the barrier for HT, or k_{HT}, only[5].

**^{1}H, 4%**	4% sucrose effect on $k_{HT,i}$, only			4% sucrose effect $k_{SP,i}$, only		
i	$k^{1H}_{obs,i}$ (s^{-1})	$k^{1H}_{SP,i}$ (s^{-1})*	$r^{1H}_{k,i}$	$k^{1H}_{obs,i}$ (s^{-1})	$k^{1H}_{SP,i}$ (s^{-1})†	$r^{1H}_{k,i}$†
10%, ↑	6.35×10^{-3}	7.24×10^{-3}	0.14	6.35×10^{-3}	6.35×10^{-3}	0
50%, ↑	3.11×10^{-3}	3.99×10^{-3}	0.28	3.11×10^{-3}	3.11×10^{-3}	0
peak	7.00×10^{-4}	1.39×10^{-3}	0.99	7.00×10^{-4}	7.00×10^{-4}	0
50%, ↓	4.30×10^{-5}	2.70×10^{-4}	5.3	4.30×10^{-5}	4.30×10^{-5}	0
10%, ↓	7.71×10^{-6}	2.89×10^{-5}	2.7	7.71×10^{-6}	7.71×10^{-6}	0

**^{2}H, 4%**	4% sucrose effect on $k_{HT,i}$, only			4% sucrose effect $k_{SP,i}$, only		
i	$k^{2H}_{obs,i}$ (s^{-1})	$k^{2H}_{SP,i}$ (s^{-1})*	$r^{2H}_{k,i}$	$k^{2H}_{obs,i}$ (s^{-1})	$k^{2H}_{SP,i}$ (s^{-1})	$r^{2H}_{k,i}$
10%, ↑	2.70×10^{-4}	7.24×10^{-3}	26	2.70×10^{-4}	6.35×10^{-3}	23
50%, ↑	1.29×10^{-4}	3.99×10^{-3}	30	1.29×10^{-4}	3.11×10^{-3}	23
peak	4.18×10^{-5}	1.39×10^{-3}	32	4.18×10^{-5}	7.00×10^{-4}	17
50%, ↓	4.48×10^{-6}	2.70×10^{-4}	59	4.48×10^{-6}	4.30×10^{-5}	10
10%, ↓	3.99×10^{-7}	2.89×10^{-5}	73	3.99×10^{-7}	7.71×10^{-6}	20

* Value for ^{1}H, 2% sucrose condition, incorporating assumption that 4% sucrose does not alter $k^{1H}_{SP,i}$, relative to 2% sucrose.

† The condition, $k^{1H}_{obs,i}$ = $k^{1H}_{SP,i}$, entails $r^{1H}_{k,i}$<<1, and therefore, that $r^{1H}_{k,i}$ is negligible.

SI References

(1) Faust, L. P.; Connor, J. A.; Roof, D. M.; Hoch, J. A.; Babior, B. M. AdoCbl-Dependent Ethanolamine Amino-Lyase from Salmonella Typhimurium. *J. Biol. Chem* **1990**, *265*, 12462–12466.

(2) Faust, L. P.; Babior, B. M. Overexpression, Purification, and Some Properties of the AdoCbl-Dependent Ethanolamine Ammonia-Lyase from Salmonella Typhimurium. *Archives of Biochemistry and Biophysics* **1992**, *294* (1), 50–54.

(3) Wang, M.; Zhu, C.; Kohne, M.; Warncke, K. Resolution and Characterization of Chemical Steps in Enzyme Catalytic Sequences by Using Low-Temperature and Time-Resolved, Full-Spectrum EPR Spectroscopy in Fluid Cryosolvent and Frozen Solution Systems. In *Methods in Enzymology*; Elsevier, 2015; Vol. 563, pp 59–94.

(4) Austin, R. H.; Beeson, K. W.; Eisenstein, L.; Frauenfelder, H.; Gunsalus, I. C. Dynamics of Ligand Binding to Myoglobin. *Biochemistry* **1975**, *14* (24), 5355–5373.

(5) Kohne, M.; Li, W.; Zhu, C.; Warncke, K. Deuterium Kinetic Isotope Effects Resolve Low-Temperature Substrate Radical Reaction Pathways and Steps in B_{12}-Dependent Ethanolamine Ammonia-Lyase. *Biochemistry* **2019**, *58* (35), 3683–3690.
